# Morphometric Analysis of the Calcaneus in a Southeast Asian Population

**DOI:** 10.7759/cureus.58899

**Published:** 2024-04-24

**Authors:** Don Koh, Beatrice Tan, Kinjal Mehta, James Loh, Le Roy Chong, Charles Kon Kam King

**Affiliations:** 1 Orthopedic Surgery, SingHealth, Changi General Hospital, Singapore, SGP; 2 Diagnostic Radiology, SingHealth, Changi General Hospital, Singapore, SGP

**Keywords:** orthopaedic trauma surgery, orthopaedic surgery, foot and ankle fracture, calcaneus fractures, calcaneum fractures

## Abstract

Introduction

The calcaneus is the most commonly fractured tarsal bone, accounting for up to 60% of tarsal bone fractures and 2% of all fractures in the body. With the calcaneus playing an important role in maintaining a stable and efficient bipedal gait, the sequelae of these injuries have also been associated with potential long-term disability or discomfort, especially if improperly managed. Incorrectly sized implants similarly cause their own set of complications, such as poor fixation, impingement, or implant prominence. This potentially increases the need for revision surgery or implant removal, with increased morbidity for the patient. As such, a thorough understanding of calcaneal morphology is vital to ensure optimal conservative and surgical management of calcaneal pathology. CT imaging has become an indispensable tool in the evaluation of such a complex three-dimensional structure and allows us to accurately map out calcaneal morphology. This study aims to evaluate calcaneal morphology in the Southeast Asian population using CT imaging and to determine if morphological differences exist between male and female patients.

Methods

Calcaneus measurements were taken from CT scans of 100 patients with intact calcanei, consisting of 34 female and 66 male patients. Patients who have had fractures or previous calcaneus surgery were excluded. IBM SPSS Statistics for Windows, Version 28.0 (Released 2021; IBM Corp., Armonk, NY, USA) was used for statistical calculations. Mean values were calculated, and t-tests were performed to establish any significant differences between measurements taken from male and female patients. Results were deemed to have a significant difference if the p-value was less than 0.05.

Results

Males had larger calcanei measurements than females in all parameters included. Calcaneal length in females measured on CT axial views was 66.2 mm, compared to 75.2 mm in males (p < 0.001). Calcaneal height, measured at the medial wall, was 28.2 mm in females and 33.9 mm in males (p < 0.001). Calcaneal height measured at the lateral wall was 33.3 mm and 38.1 mm in females and males, respectively (p > 0.001). Calcaneal width was 33.0 mm in females and 36.9 mm in males (p < 0.001). The mean dimensions measured in the total sample were an axial length of 72.1 mm, a medial wall height of 32.0 mm, a lateral wall height of 36.4 mm, and a width of 35.6 mm.

Conclusion

There is a significant difference in calcaneal morphology on CT imaging between male and female patients in the Southeast Asian population, which is an important consideration for surgical planning and the selection of appropriately sized implants.

## Introduction

The calcaneus is the greatest tarsal bone and plays an important function in stabilizing and maintaining an efficient bipedal gait [1]. The foot bones are involved in 10% of all fractures, of which the calcaneus is the most commonly affected, representing 2% of all fractures in the body and 60% of all tarsal bone fractures [2,3].

Up to 75% of all calcaneal fractures were shown to be intra-articular [4], and while simple fractures can be approached using minimally invasive techniques, more complex fractures often require fixation with plate and screw constructs via open surgery [5]. The use of correctly sized implants is crucial to achieving adequate fixation as well as avoiding implant prominence, which can lead to mechanical impingement and soft tissue complications such as wound necrosis [6].

Radiographic investigations are essential in the assessment of calcaneal fractures. CT imaging has now become an indispensable tool in providing three-dimensional morphological information for the treatment of this complex structure [7,8]. CT imaging has been previously utilized to develop an anatomically accurate atlas of the calcaneus [9], and various studies have already shown individual variations in calcaneal morphometry [10].

In order to ensure optimal treatment of patients with calcaneal fractures, as well as reduce the need for revision surgery or implant removal, a thorough understanding of calcaneal morphology is vital. The aim of this paper is therefore to evaluate the calcaneal morphology in the Southeast Asian population for gender differences using CT imaging to provide more information for surgical planning.

## Materials and methods

A convenience sample study was carried out by analyzing the CT foot scans of patients with intact calcanei at SingHealth, Changi General Hospital, Singapore, Singapore. All patients who attended our hospital and met the eligibility criteria were included in this study.

Exclusion criteria included patients who were skeletally immature (taken as aged 18 and below), as well as patients who had suffered previous fractures or had undergone surgery to the measured calcaneus.

This study was approved by the SingHealth Institutional Review Board (approval number 2016/3086).

Radiographic parameters

**Figure 1:**
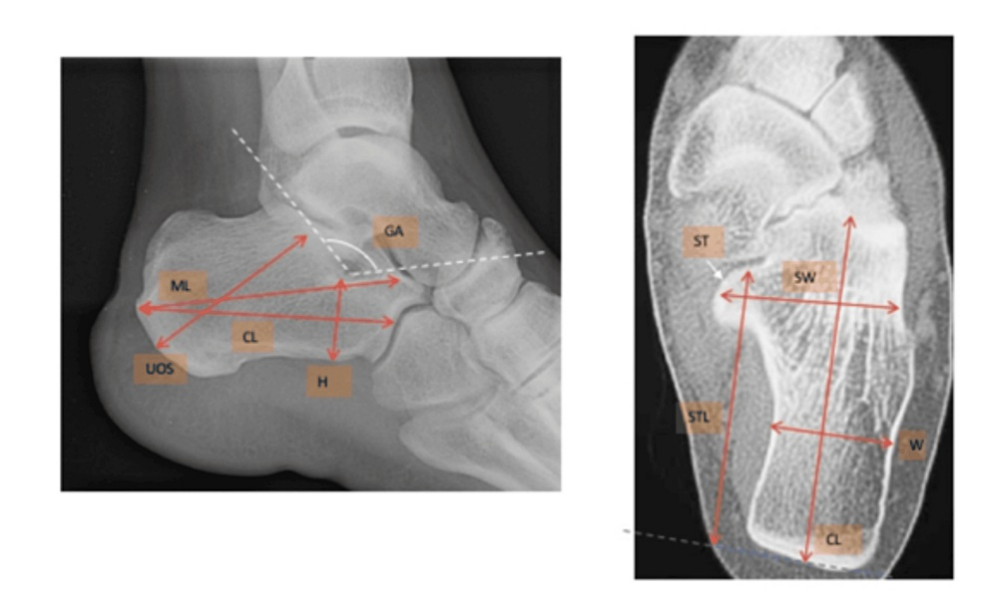
Illustration of measured parameters Sagittal view (left) and axial view (right) of the calcaneus and measured parameters CL: calcaneal length along the medial wall (linear distance between the center of the vertical surface of the cuboid facet and the posterior point of the calcaneal tuberosity along the midline of the calcaneus); CL: calcaneal length at the midline (linear distance between the center of the vertical surface of the cuboid facet and the posterior point of the calcaneal tuberosity along the midline of the calcaneus); GA: the angle measured at the intersection of the posterior facet and the line along the superior surface of the anterior process; H: height at midline from Gissane angle (linear distance between the most inferior point on the calcaneus body to Gissane angle at midline); ML: maximum length (linear distance between the most posterior point on the calcaneus tuberosity and the most anterior talar point); ST: sustenaculum tali; STL: (maximum length till sustentaculum tali) linear distance between the most posterior point on the calcaneus tuberosity and the most anterior point of sustenaculum tali; SW: width from lateral to medial screw trajectory (linear distance between the most lateral point of the posterior articular facet and most medial point of sustenaculum tali); UOS: upward oblique screw trajectory to posterior facet (linear distance between the midpoint of posterior and inferior calcaneus tuberosity to the midpoint of the posterior facet); W: width from lateral to medial screw trajectory (linear distance between medial and lateral surfaces of the calcaneus body)

Morphology parameters were measured using computer software by snapping a cursor on anatomical landmarks that were previously described by Qiang et al. for calcaneal morphological characteristics [11]. Measurements were performed by a single author, and the mean values for each parameter were taken. All parameters were measured on either the sagittal or axial planes. There were a total of 14 parameters, 12 linear distances, and two angles. A detailed description of the landmarks and methods used for the measurement of the parameters is listed below in Table 1 and illustrated in Figure 1.

**Table 1:** Description of landmarks and methods used for parameter measurements CL: calcaneal length along the medial wall (linear distance between the center of the vertical surface of the cuboid facet and the posterior point of the calcaneal tuberosity along the midline of the calcaneus); CL: calcaneal length at the midline (linear distance between the center of the vertical surface of the cuboid facet and the posterior point of the calcaneal tuberosity along the midline of the calcaneus); GA: the angle measured at the intersection of the posterior facet and the line along the superior surface of the anterior process; H: height at midline from Gissane angle (linear distance between the most inferior point on the calcaneus body to Gissane angle at midline); ML: maximum length (linear distance between the most posterior point on the calcaneus tuberosity and the most anterior talar point); STL: (maximum length till sustentaculum tali) linear distance between the most posterior point on the calcaneus tuberosity and the most anterior point of sustenaculum tali; SW: width from lateral to medial screw trajectory (linear distance between the most lateral point of the posterior articular facet and most medial point of sustenaculum tali); UOS: upward oblique screw trajectory to posterior facet (linear distance between the midpoint of posterior and inferior calcaneus tuberosity to the midpoint of the posterior facet); W: width from lateral to medial screw trajectory (linear distance between medial and lateral surfaces of the calcaneus body)

Parameter		Description
Calcaneal length along	Medial wall (a)	The linear distance between the center of the vertical surface of the cuboid facet and the posterior point of the calcaneal tuberosity along the (a) medial wall of the calcaneus
	Lateral wall (b)	(b) Lateral wall of the calcaneus
	Midline (c)	(c) Midline of the calcaneus (CL)
Maximum length (a)		Linear distance between the most posterior point on the calcaneus tuberosity and the most anterior talar point (ML)
Maximum length till sustentaculum tali (b) (measured on axial view)		Most anterior point of the sustentaculum tali (STL)
Height at midline from	Gissane angle (a)	The linear distance between the most inferior point on the calcaneus body and the Gissane angle (GA) (the angle measured at the intersection of the posterior facet and the line along the superior surface of the anterior process) at the midline (H)
	Midline parallel to the calcaneocuboid joint (b)	The most superior surface at the midline, parallel to the calcaneocuboid joint
	Medial wall of calcaneus (c)	The most superior surface at the medial wall
	Lateral wall of calcaneus (d)	The most superior surface at the lateral wall
Width from	Lateral to medial screw trajectory (a)	The linear distance between the most lateral point of the posterior articular facet and the most medial point of the sustenaculum tali (SW)
	Lateral to medial widths (b)	Linear distance between the medial and lateral surfaces of the calcaneus body (W)
Upward oblique screw trajectory to posterior facet (measured in sagittal view)		Linear distance between the midpoint of the posterior and inferior calcaneus tuberosities to the midpoint of the posterior facet (UOS)

Statistical analysis

IBM SPSS Statistics for Windows, Version 28.0 (Released 2021; IBM Corp., Armonk, NY, USA) was used for statistical calculations. Mean values were calculated, and t-tests were performed to establish any significant differences between measurements taken from male and female patients. Quantitative results are reported as mean (SD) with 95% CI. Results were considered to be statistically significant if the p-value was less than 0.05. An independent statistician performed all analyses.

## Results

A total of 100 patients were included in our study: 34 females and 66 males. Males had larger calcanei measurements than females in all parameters included. A detailed summary of the results is displayed in Table 2 below.

**Table 2:** Morphometric measurement of the calcaneus All measurements were presented as mean (SD).

Measurement	All (n = 100)	Female (n = 34)	Male (n = 66)	p-value
Calcaneal length measured (mm)				
Medial wall	60.8 (6.1)	54.6 (3.8)	64.0 (4.2)	<0.001
Lateral wall	70.6 (6.1)	64.1 (4.4)	73.9 (3.8)	<0.001
Midline	70.8 (6.0)	64.7 (4.3)	74.0 (3.9)	<0.001
Calcaneal length measured on axial view (mm)				
Maximum length	72.1 (6.0)	66.2 (4.3)	75.2 (4.3)	<0.001
Maximum length till sustentaculum tali	55.4 (5.8)	51.5 (4.7)	57.4 (5.2)	<0.001
Height (mm)				
At midline, from the Gissane angle	39.4 (4.0)	35.4 (2.3)	41.5 (3.0)	<0.001
At midline, parallel to the calcaneocuboid joint	22.0 (2.9)	19.8 (2.1)	23.1 (2.6)	<0.001
At the medial wall of the calcaneus	32.0 (4.2)	28.2 (3.9)	33.9 (3.0)	<0.001
At the lateral wall of the calcaneus	36.4 (3.7)	33.3 (2.6)	38.1 (3.1)	<0.001
Width (mm)				
Lateral to medial screw trajectory	40.9 (5.1)	36.9 (3.6)	42.9 (4.6)	<0.001
Lateral to medial of the calcaneal body	35.6 (3.9)	33.0 (3.5)	36.9 (3.5)	<0.001
Sagittal view (mm)				
Upward oblique screw trajectory to the posterior facet	50.8 (4.8)	46.4 (3.2)	53.1 (3.7)	<0.001

Calcaneal length

The maximum calcaneal length measured on axial view in females was 66.2 mm, compared to 75.2 mm in males (p < 0.001). Calcaneal length measured in the midline in females was 64.7 mm and 74.0 mm in males (p < 0.001).

Calcaneal height

Calcaneal height, measured at the medial wall, was 28.2 mm in females and 33.9 mm in males (p < 0.001). Calcaneal height measured at the lateral wall was 33.3 mm and 38.1 mm in females and males, respectively (p > 0.001).

Calcaneal width

Calcaneal body width was 33.0 mm in females and 36.9 mm in males (p < 0.001). The width of the lateral to medial screw directory was 36.9 mm in females and 42.9 mm in males (p < 0.001).

Upward oblique screw trajectory to the posterior facet

The measured length for the upward oblique screw trajectory to the posterior facet was 46.4 mm in females and 53.1 mm in males (p < 0.001).

Mean dimensions

The mean dimensions measured in the total sample were an axial length of 72.1 mm, a medial wall height of 32.0 mm, a lateral wall height of 36.4 mm, and a width of 35.6 mm.

## Discussion

There are significant differences between male and female calcaneal morphology in the Southeast Asian population based on the measured parameters in our study. Our findings are in keeping with existing literature, which has shown that not only are there gender differences in calcaneal morphology, but these differences are also stark enough to serve as a reliable criterion for sex determination [12-14]. The significance of this information has also led to its application in other fields, such as forensic pathology. Furthermore, males having large calcaneal dimensions are also in line with previous studies that have evaluated gender differences across other geographical areas [12,15].

Apart from parameters of the calcaneus, such as Boehler angle and maximum height of the calcaneal body, which have been evaluated by previous studies, our study also includes surgery-specific measurements [16,17]. Measurements of the length of the upward oblique screw trajectory to the posterior facet and the lateral to medial screw trajectory to the sustentaculum tali were also shown in our paper to be significantly different between the genders. Other morphological studies have also shown variations in specific anatomical structures between genders and the thickening of the sustentaculum tali with age [18]. Having knowledge of the general lengths of the trajectories used for screw placement provides the surgeon with a better gauge for implant choices.

While the female calcanei have consistently been reported to have smaller dimensions, an important consideration is that angular parameters appear to remain comparable. Implant plates have therefore been designed to provide serial increments in dimensions to provide adequate options during surgery [19]. However, consideration must be made for specific differences in calcaneal morphology in each local population. As imaging and statistical shape modeling advance, further studies can be performed to compare independent variations in calcaneal morphology between not just genders but also populations. This information can be used to develop gender- and population-specific implants for a better anatomical fit [20].

One of the limitations of the study is the lack of control for the comparison of morphology with the contralateral calcaneus. Knowledge of the symmetry between both calcanei is crucial, as the unaffected side can serve as a reference guide for implant sizes and position when treating a deformed calcaneus, such as in cases of trauma [21-23]. Existing literature has shown that contralateral calcanei generally display symmetry in terms of the shapes and number of articular facets [12]. However, specific measurements such as the transverse width of the calcaneus and the sulcus length and width have been reported to be asymmetric [24,25].

Moving forward, further studies should also include comparisons for differences in laterality in the Southeast Asian population itself, as ethnic variations in calcaneal morphology have been known to be present [26]. Furthermore, our study was also limited by the small sample size of 100 patients.

To our knowledge, this is the first paper describing the morphological characteristics with comparison between genders in the Southeast Asian population, which provides pertinent information for making appropriate surgical decisions.

## Conclusions

There is a significant difference in calcaneal dimensions between female and male patients in the Southeast Asian population. Males have larger dimensions with regard to calcaneal length, height, and width, as well as lengths for screw placement. Gender differences should be carefully considered when selecting implants during surgical planning to ensure appropriate sizing.
